# IFNIKB: a type I interferon database for antitumour immunity studies

**DOI:** 10.1093/database/baag053

**Published:** 2026-09-07

**Authors:** Fubo Ma, Kang Li, Yangchao Yu, Lei Zhang, Liguo Zhang, Bing Li, Le Zhang

**Affiliations:** College of Computer Science, Sichuan University, Chengdu, 610065, China; West China Biomedical Big Data Center, West China Hospital, Sichuan University, Chengdu, 610041, China; West China Biomedical Big Data Center, West China Hospital, Sichuan University, Chengdu, 610041, China; College of Computer Science, Sichuan University, Chengdu, 610065, China; College of Computer Science, Sichuan University, Chengdu, 610065, China; Key Laboratory of Biomacromolecules (CAS), National Laboratory of Biomacromolecules, CAS Center for Excellence in Biomacromolecules, Institute of Biophysics, Chinese Academy of Sciences, Beijing, 100101, China; College of Computer Science, Sichuan University, Chengdu, 610065, China; College of Computer Science, Sichuan University, Chengdu, 610065, China

## Abstract

Type I interferon (IFN-I) is an important class of cytokines that can inhibit tumour progression through mechanisms such as immunomodulation of the tumour microenvironment or targeting cellular components. Although various endogenous and exogenous IFN-I therapeutic strategies have been developed, reports of immune cell dysfunction resulting from IFN-I signal enhancement indicate that optimal strategies have yet to be established. However, heterogeneous data of IFN-I are currently spread across multiple public databases and lack systematic integration, which poses challenges for knowledge acquisition and clinical research promotion. Herein, we develop the IFN-I Knowledge Base (IFNIKB), the first specific database for IFN-I. It integrates 26 273 literature articles on IFN-I antitumour immunity, 2202 clinical trial records, and data on 8372 genes and 7164 proteins across 654 species. Furthermore, we design an automated workflow for knowledge discovery from literature. Users can construct on-demand knowledge graphs to explore entities and relationships through interactive visualizations, temporal trends, and network topology analyses. Additionally, we provide tools for multiple sequence alignment, sequence identity computation, and phylogenetic analysis to interpret IFN-I from molecular perspectives. Therefore, researchers can employ IFNIKB to conveniently acquire knowledge, propose hypothesis, optimize experimental design, and identify potential clinical therapeutic targets. **Database URL:**  http://www.combio-lezhang.online/IFNIKB/home

## Introduction

Type I interferon (IFN-I) is a family of proinflammatory cytokines [1, 2], which could be identified in all lineages of normal or malignant cells during the infection of pathogens [3]. Multiple reports indicate that both endogenous and exogenous IFN-I [4] can directly or indirectly inhibit tumour growth through cancer cell-intrinsic effects [2](such as the upregulation of immune-interacting molecules [5–7] and control of functional genes [8–10]) and immunomodulatory effects [2] (such as promoting the activity of immune cells [11–13] and inhibiting immune-suppressive cells [14, 15]). Therefore, the α2 subtype of human IFN-I was prescribed as the first clinical drug for cancer immunotherapy by US Food and Drug Administration (FDA) due to its inhibition of cell proliferation [3, 16, 17]. However, chronic and weak IFN-I responses in cancer may cause negative effects, including cellular dormancy, incomplete elimination of cancer cells [18, 19], as well as providing proinflammatory mediators that promote tumour progression [20, 21]. Evidently, maximizing the antitumour effects of IFN-I [2] requests interdisciplinary and long-term research focus.

Although the background above highlights the complexity and diversity of the IFN-I signalling network within the tumour microenvironment [22, 23], this large amount of literature has not received sufficient support from emerging bioinformatics technologies [24–31]. On one hand, heterogeneous data, such as literature reports, clinical trial cohorts, and sequencing datasets, are fragmented across multiple public databases like PubMed [32], ClinicalTrials.gov [33], and NCBI Gene [34], but a domain-specific database for their systematic integration has yet to be established. On the other hand, although Rusinova *et al*. have developed a workflow for the retrieval and analysis of interferon-stimulated gene (ISG) data [35], an automated workflow is currently lacking for retrieving, analyzing, and visualizing biomedical entities and relationships of interest from IFN-I antitumour immunity literature reports. This situation not only causes obstacles in knowledge acquisition and scientific discovery, but also fails to achieve the objective of data-driven discovery in biology [36, 37] and medicine [38].

For these reasons, we develop the IFN-I Knowledge Base (IFNIKB) to address these limitations. First, IFNIKB integrates heterogeneous IFN-I data from multiple databases [32–34, 39, 40], which represents the first IFN-I-specific database, to the best of our knowledge. Second, we provide the ‘Antitumour Immunity Reports’ module, which introduces an automated workflow that transforms ∼26 000 literature reports on IFN-I antitumour immunity into interactive knowledge graphs [41]. Third, the ‘Clinical Applications’ and ‘Molecular Information’ modules we designed provide user-friendly search functionalities for ∼2200 IFN-I clinical trial records and for ∼650 species, 8400 genes, as well as associated proteins, respectively, which is the most comprehensive knowledge base currently available for this domain. Furthermore, within the ‘Evolution & Comparative Genomics’ module, we support molecular evolution studies and guide experimental design by integrating three IFN-I evolutionary analysis tools: multiple sequence alignment (MSA) [29, 42], sequence identity matrix [43, 44], and phylogenetic tree construction [45, 46]. Finally, we provide a hypothetical case study integrating the four main modules of IFNIKB above, which is developed to help IFN-I scientists for cutting-edge scientific research and hypotheses proposing. In summary, IFNIKB will effectively increase the accessibility of IFN-I knowledge in fields of biology and medicine.

## Methods

### Data collection and preprocessing

**Figure 1:**
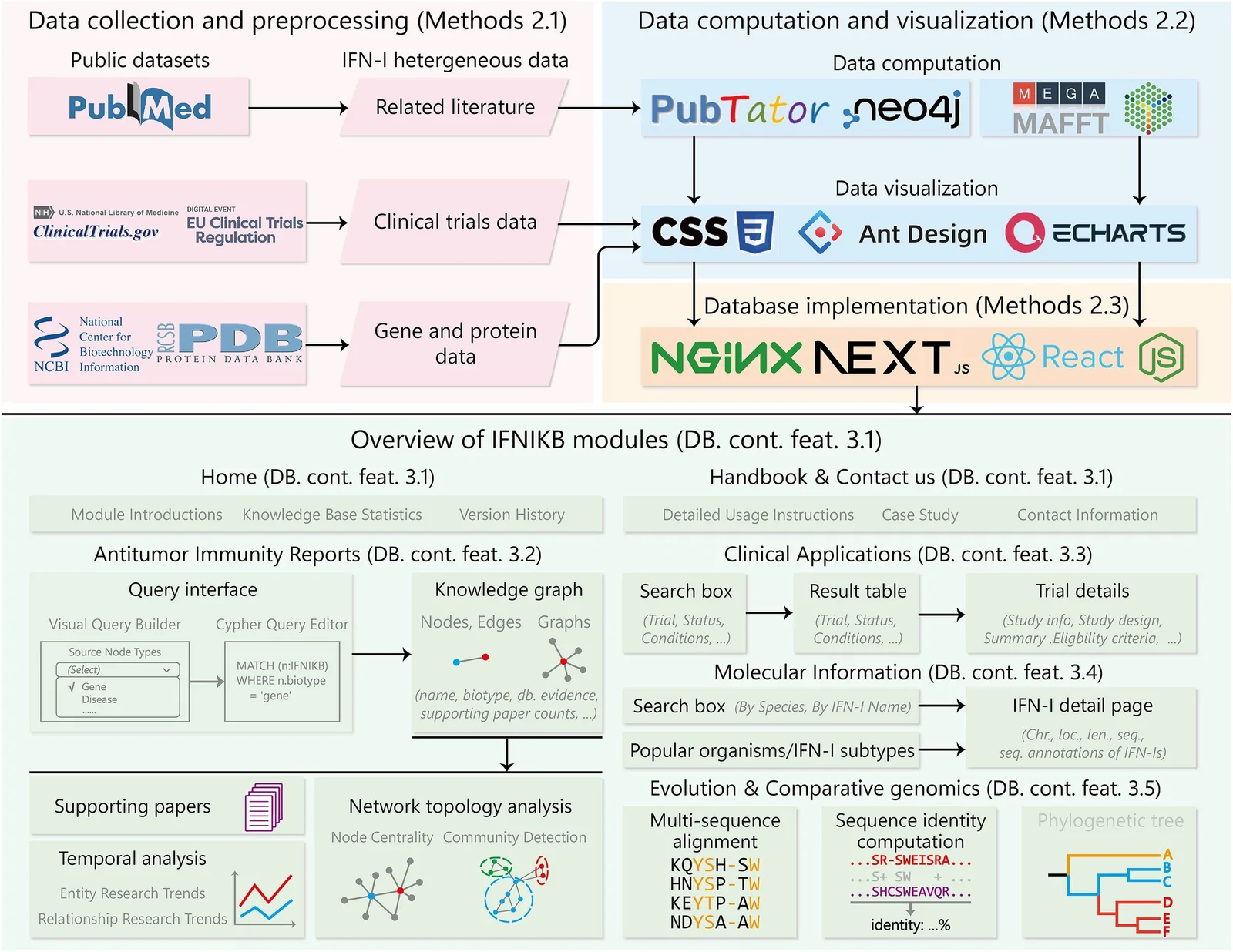
The construction and workflow of IFNIKB. In the figure, three regions in the upper portion represent three steps of database construction, which correspond respectively to parts of the ‘Methods’ section; one region in the lower portion represents four modules of IFNIKB database workflow, which correspond respectively to parts of the ‘Database content and features’ section.

We systematically collect and process data from three primary sources. First, we curate a corpus of 26 273 research articles from NCBI databases by employing a multi-step query and filtering strategy (Method S1) [32]. Second, we acquire 2202 clinical trial records by querying the ClinicalTrials API [33] and the EU Clinical Trials Register [39], followed by a rigorous screening and manual curation process to ensure data relevance and consistency (Table S1). Third, we retrieve 8372 IFN-I genes and 7164 corresponding protein sequences from 654 species via the NCBI Gene database [34], establishing a foundational dataset for comparative genomics analysis (Fig. 1).

### Data computation and visualization

A comprehensive IFN-I antitumour immunity knowledge graph is constructed by extracting biomedical entities and their relationships from the collected literature using PubTator3 [47]. Entities were normalized to controlled vocabularies and stored with stable node IDs: MeSH (chemicals and most diseases), OMIM (a minority of diseases), NCBI Gene (genes), NCBI Taxonomy (species), Cellosaurus (cell lines), and LitVar (variants) (Table S2). Mentions mapped to the same vocabulary identifier were merged into a single node, and only PubTator3-defined relation types were retained. We performed automated quality control (QC), including biotype–identifier consistency checks, vocabulary-based validations, and removal of relationships with missing endpoints, self-loops, or undefined types (Table S2). After QC, the graph comprising 14 466 entities and 59 222 relationships (from 14 531 entities and 60 150 relationships before QC; Table S2) is stored and managed in a Neo4j graph database [48]. We perform downstream analyses on this graph, including temporal trend analysis, node centrality analysis, and community detection, to uncover key research patterns and influential entities (Method S2). Furthermore, we integrate a suite of comparative genomics tools for multiple sequence alignment, sequence identity calculation (MEGA [43]), and phylogenetic analysis (TimeTree [45]).

All data and analysis results are integrated into a responsive web portal. The user interface is developed using the Ant Design [49] component library, and interactive data visualizations, including networks, charts, and genomic maps, are rendered using Apache ECharts [50].

### Database implementation

IFNIKB is developed using Next.js (v13.0.6) as the main web framework. The frontend interface employs React (v18.2.0) [51] as the core library to build up high-quality interface components. IFNIKB is deployed on the CentOS (v7.0) operating system [52], as well as reverse proxy and static resource caching are served by the Nginx (v1.18.0) server [53]. API services are supported by the Node.js (v23.11.0) environment. IFNIKB has been tested with major browsers such as Google Chrome, Microsoft Edge, and Safari, as well as mobile devices including smartphones and tablets, which guarantees broad compatibility across various client environments.

## Database content and features

### Overview of IFNIKB modules

IFNIKB consists of six modules (Fig. 1). The ‘Home’ page provides statistical summaries and navigation, while a ‘Handbook’ page offers detailed user guidance. Beside these two supporting modules, IFN-I-related literature reports, clinical trial records, gene and protein information, and comparative genomics tools are provided by the ‘Antitumour Immunity Reports’ module, ‘Clinical Applications’ module, ‘Molecular Information’ module, and ‘Evolution & Comparative Genomics’ module, respectively (Fig. 1). These four modules, detailed in the following sections, collectively develop an integrated platform for IFN-I data exploration, analysis, and hypothesis generation.

### Antitumour Immunity Reports module

We design the ‘Antitumour Immunity Reports’ module for literature about IFN-I antitumour immunity. Since this module integrates comprehensive reports from public databases, extracts biomedical entities and their relationships from the literature, and constructs the first IFN-I antitumour immunity knowledge graph (Methods 2.1.1 and 2.2), it could facilitate user queries on relationships between entities on interested topics.

**Figure 2:**
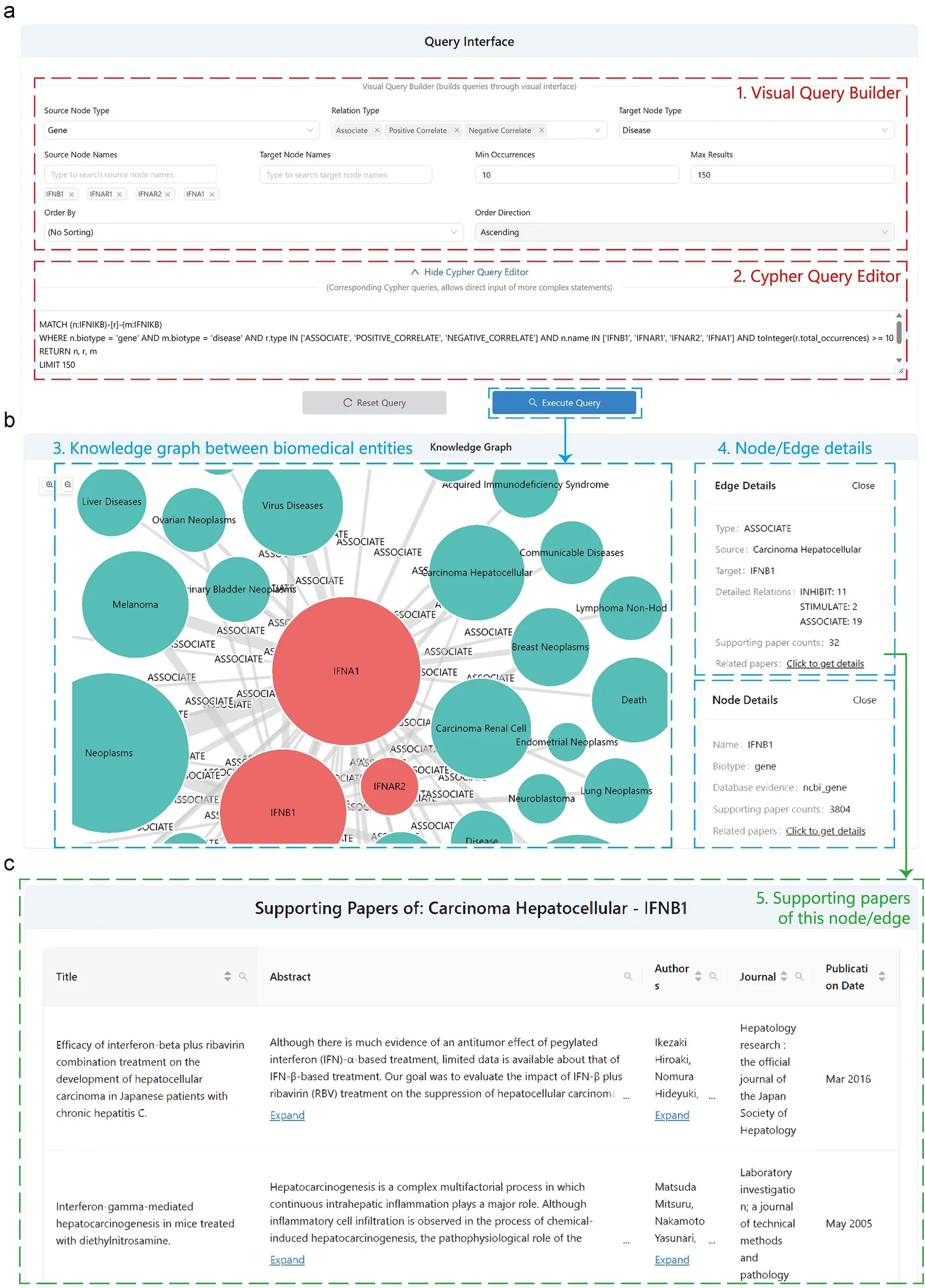
Performing knowledge query by the automated workflow within the IFN-I antitumour immunity knowledge graph. (a) The ‘Query Interface’ component. (b) The ‘Knowledge Graph’ component with the pop-up window. Entities (nodes) and relationships (edges) are represented by circles and gray lines in the graph, respectively. Note that only one pop-up window is displayed at a time in this interface, but we use image composition for the illustrative purpose. (c) The ‘Supporting Papers’ component.

The first step of the workflow to retrieve literature knowledge from the knowledge graph is data query, which generates Cypher queries via the interactive interface within the ‘Visual Query Builder’ (Box 1 of Fig. 2a). Our ‘Visual Query Builder’ supports filter criteria such as ‘Relation Type’ (e.g. ‘Associate’), ‘Source/Target Node Type’ (e.g. ‘Gene’), and specific ‘Source/Target Node Names’ (e.g. IFNB1). Additionally, IFNIKB allows users to directly input complex Cypher query statements into the ‘Cypher Query Editor’ to obtain more precise search results (Box 2 of Fig. 2a).

**Figure 3:**
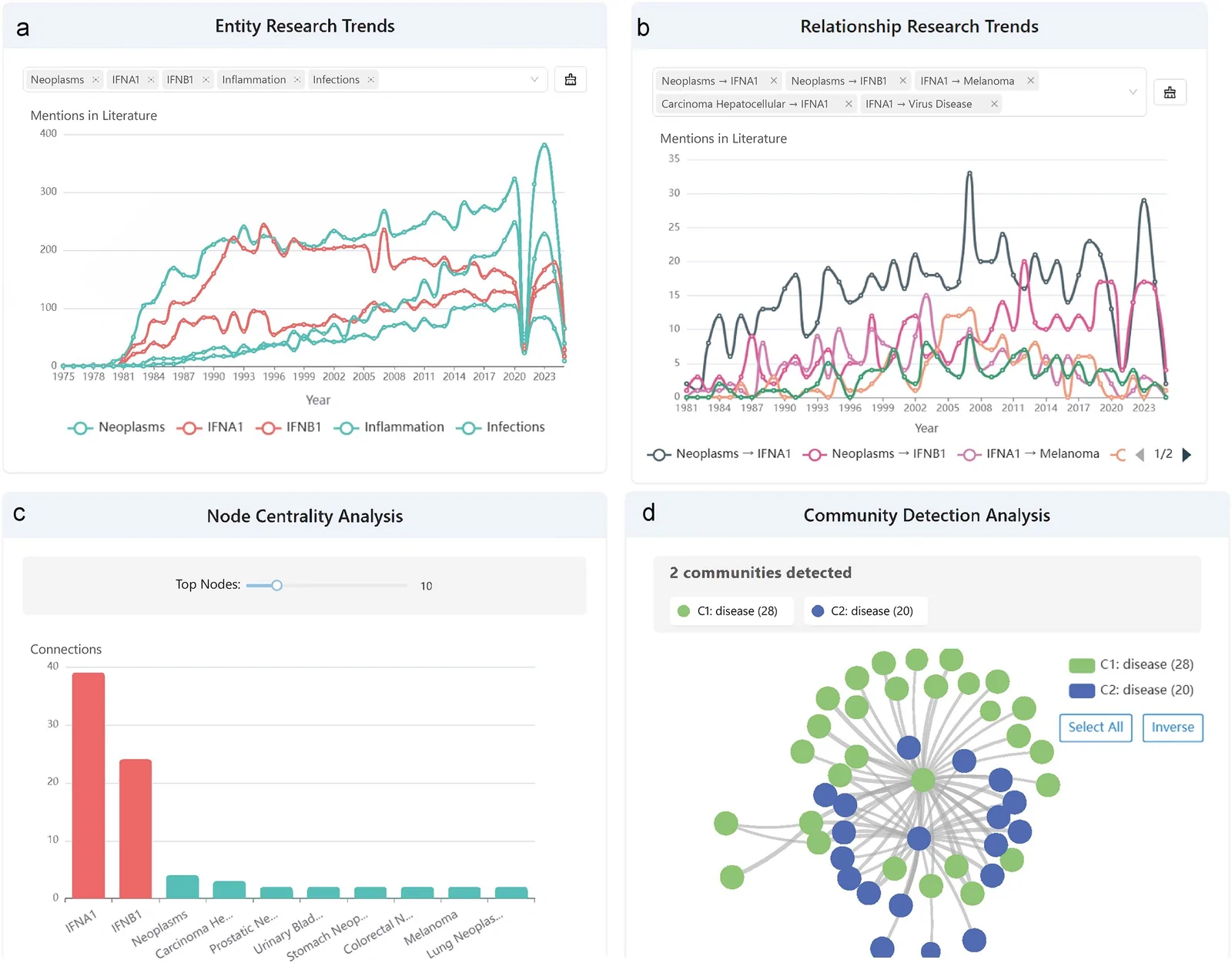
Analyses of time-series and network topology facilitate IFN-I antitumour immunity knowledge reasoning. (a) The ‘Entity Research Trend’ component. (b) The ‘Relationship Research Trend’ component. (c) The ‘Node Centrality Analysis’ component. (d) The ‘Community Detection Analysis’ component.

Upon execution of a data query, our workflow automatically generates the corresponding graph results (Fig. 2b), displays the supporting papers (Fig. 2c), as well as initiates downstream computings (time-series analysis and network topology analysis) and visualization rendering (Fig. 3). Here, users can click on the nodes and edges of generated knowledge graph (Box 3 of Fig. 2b) to view the detailed information of biomedical entities or relationships, which includes information such as ‘Name (e.g. IFNB1), ‘Biotype’ (e.g. gene), and ‘Database evidence’ (e.g. ncbi_gene) of the entity, and ‘Type’ (e.g. ASSOCIATE) of the relationship (Box 4 of Fig. 2b). Specifically, clicking the ‘Click to get details’ link refreshes the ‘Supporting papers’ component with the literature evidence supporting this node/edge, where users can perform further searches to retrieve literature based on specified topics, authors, journals, or publication dates (Box 5 of Fig. 2c).

We develop multiple components to support in-depth knowledge reasoning from the graph (Fig. 3). On one hand, we implement time-series analysis components to analyse and visualize the research trends of entities and relationships within the literature, which allows users to identify hot entities and relationships and quantify the evolution of research focus over time (Fig. 3a and b). For instance, by observing publication trends for genes like ‘IFNB1’ or diseases like ‘Breast Neoplasms’, users can identify related historical inflection points in immunotherapy, thereby discovering research directions with significant therapeutic potential or identifying knowledge gaps.

On the other hand, we develop network topology analysis components to locate the clusters of important entities, such as genes, diseases, and chemicals, within specific topics. The node centrality analysis identifies the key entities with the highest degree (most connections) within the knowledge network (Fig. 3c), while community detection analysis is employed to identify key nodes and densely connected entity communities, which can elucidate different gene pathways involved in IFN-I antitumour immunity (Fig. 3d). For example, ‘IFNA1’ and ‘IFNB1’ exhibit the highest degrees in the graph result, and the network forms two distinct communities centred on these nodes (Fig. 3c and d). This observation is consistent with the reported differential functions of the α and β subtypes of IFN-I in immune responses [16, 17].

By integrating knowledge graph tools, supporting manuscripts, analysis components, and visualization techniques, we have addressed the limitation whereby IFN-I literature knowledge could not be processed through an integrated ‘query-compute-analyze-present’ pipeline using classical static workflows, which not only provides the theoretical support for studying and discovering novel context-dependent mechanisms of IFN-I, but also for immunotherapies targeting IFN-I pathway design.

### Clinical Applications module

IFN-I has been applied in many clinical contexts, including antiviral and antiproliferative therapies, as well as treatments for hepatitis C and multiple sclerosis [3, 16]. To assist clinical researchers in acquiring relevant knowledge, retrieving application history, and promoting the clinical translation of IFN-I, we develop the ‘Clinical Applications module’ (Methods 2.1.2 and 2.2).

**Figure 4:**
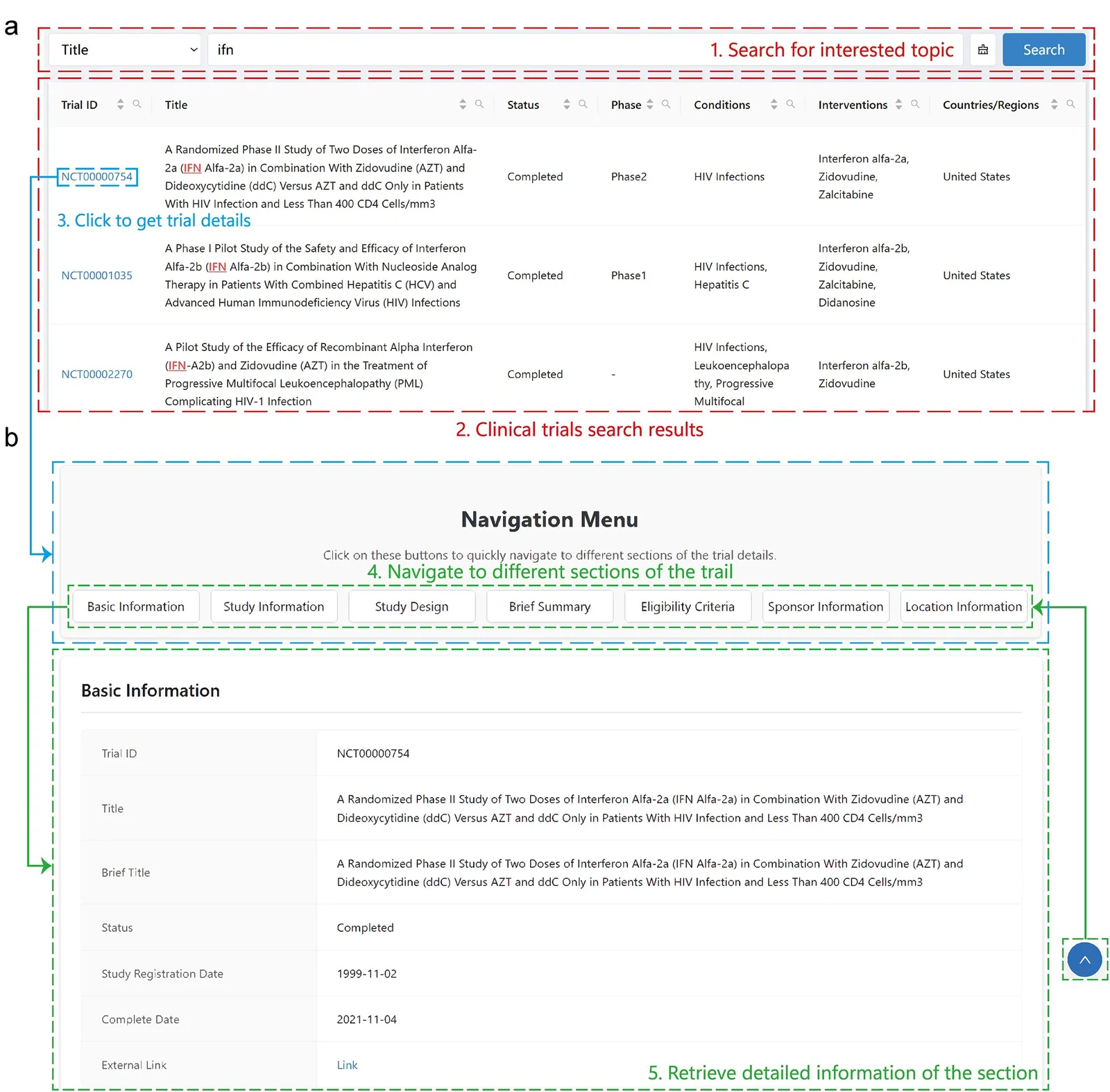
Retrieving IFN-I clinical trial records of interest via the ‘Clinical Applications’ module. (a) The clinical trial search interface. (b) The clinical trial details page.

Users can search for clinical trial records from six fields: ‘Title’, ‘Status’, ‘Conditions’, ‘Phase’, ‘Interventions’, and ‘Countries/Regions’ after accessing the module (Box 1 of Fig. 4a). Subsequently, the results Table presents a basic description of trials, and users can perform further searches within each field to refine target trial records (Box 2 of Fig. 4a).

By clicking a ‘Trial ID’ of interest within the Table, the system navigates to the trial details page (Box 3 of Fig. 4b). Here, trial records are categorized into seven sections: ‘Basic Information’, ‘Study Information’, ‘Study Design’, ‘Brief Summary’, ‘Eligibility Criteria’, ‘Sponsor Information’, and ‘Location Information’ (Box 4 of Fig. 4b). Each section is accessible via the navigation menu, where details of IFN-I clinical trial data are displayed (Box 5 of Fig. 4b). This knowledge enables users to understand the design objectives and the demographic/geographic features of enrolled participants of the trial, thereby helping researchers design and implement multi-centre clinical trials. Additionally, users can click the ‘Basic Information’ button and hyperlinks displayed under ‘External Links’ to access the original information from the source database.

### Molecular Information module

The paradigm shift driven by precision medicine has prompted users to address clinical questions from genetic and molecular perspectives [54, 55]. Therefore, in addition to the knowledge from literature reports and clinical trial records, IFNIKB offers the ‘Molecular Information’ module, which integrates the comprehensive and cross-species data of IFN-I genes and proteins (Method 2.1.3 and 2.2). This helps users to understand molecular-level differences among various IFN-I subtypes across species, in addition to the clinically prescribed IFN-α2 and IFN-β, thereby creating possibilities for developing novel clinical drugs.

**Figure 5:**
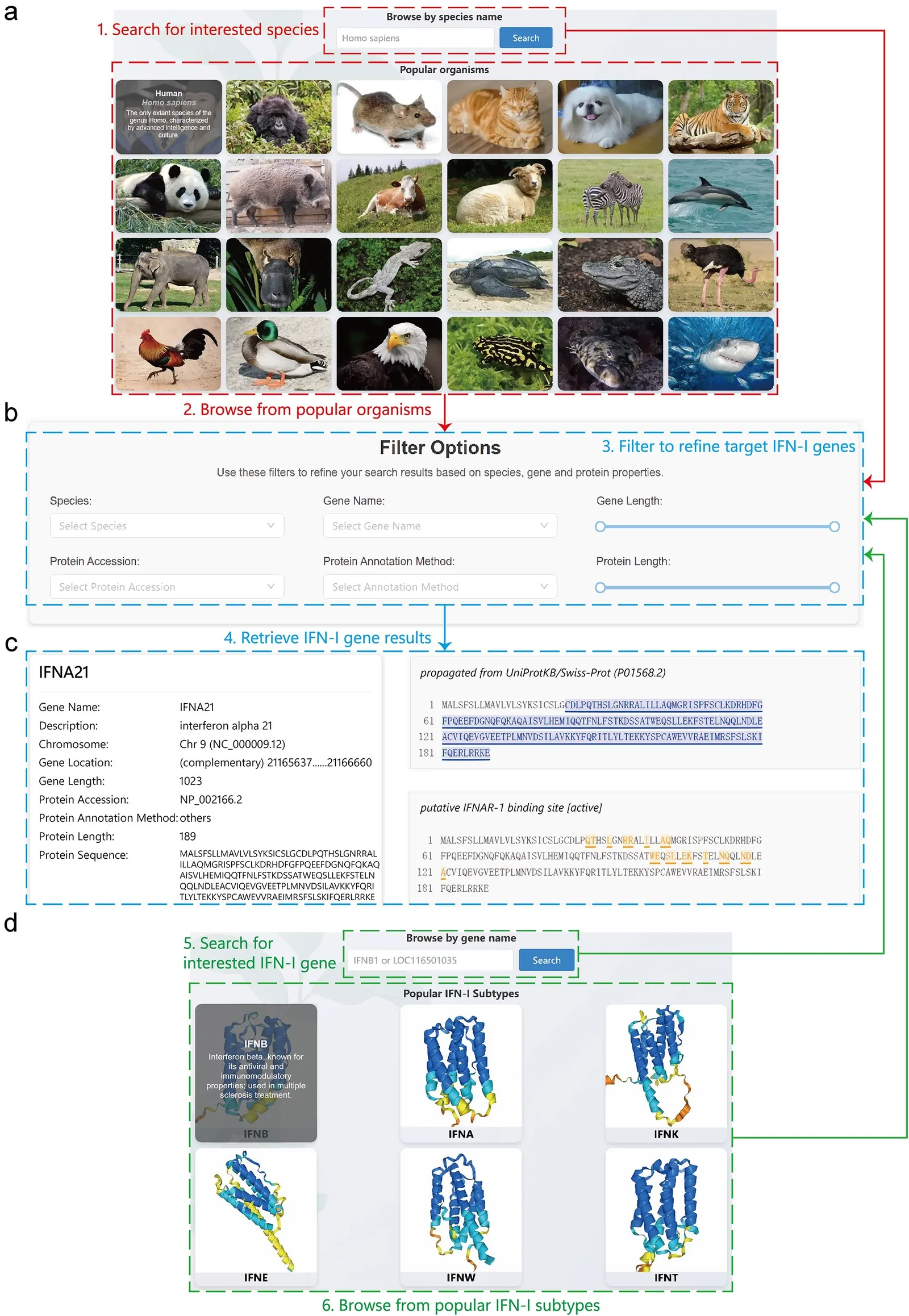
Obtaining comprehensive IFN-I gene and protein data via the ‘Molecular Information’ module. (a) The page for searching by species name. Hovering the mouse over the image of a popular organism displays a brief description of the corresponding species. (b) The filter option of detail page. (c) Information displayed on the IFN-I gene card within the detail page. Texts are under image composition for the illustrative purpose. (d) The page for searching by IFN-I gene name. Hovering the mouse over the image of a popular IFN-I subtype displays a brief description of the corresponding IFN-I subtype.

This module supports searching for IFN-I of interest by either species name or gene name. The following provides usage instructions through the former as an example. First, users can either input the Latin name of the species (e.g. *Homo sapiens*, Box 1 of Fig. 5a) into the search box or click on the image of a popular organism (Box 2 of Fig. 5a) to navigate the IFN-I gene details page of the specified species.

In response to immune evasion strategies employed by rapidly evolving pathogens, the selective pressure of host-pathogen co-evolution (the Red Queen hypothesis [56]) drives the diversification of IFN-I gene subtypes and the expansion of gene copy numbers [57]. Given that many species possess tens or even dozens of IFN-I genes, we design a ‘Filter Options’ component on the detail page to filter IFN-I results of species (Box 3 of Fig. 5b). This includes six options such as ‘Gene Name’ (e.g. IFNA1), ‘Protein Accession’ (e.g. BAM72353.1), and ‘Protein Annotation Method’ (e.g. predicted by: Gnomon). Subsequently, the search results present detailed information for each IFN-I gene in a card format (Box 4 of Fig. 5c). This includes details such as ‘Description’ (e.g. interferon alpha 21), ‘Chromosome’ (e.g. Chr 9 (NC_000009.12)), and ‘Gene Location’ (e.g. (complementary) 21 165 637…21 166 660).

Notably, the card also displays the ‘Sequence Annotations’ component we developed for IFN-I annotation, which renders specific amino acids within the protein sequence according to the annotation results (e.g. mature protein and IFNAR-1 binding site, etc., Box 4 of Fig. 5c). This facilitates the rapid localization of key structural domains within the IFN-I protein and enables the comparison of structural differences. Meanwhile, it provides a visual reference of molecular interaction sites to inform the design of functional validation experiments. Finally, users can follow the procedures described above to retrieve search results for specific IFN-I genes or IFN-I subtypes either by inputting an IFN-I gene name (Box 5 of Fig. 5d) or by clicking on the image of a popular IFN-I subtype (Box 6 of Fig. 5d).

### Evolution & Comparative Genomics module

To complement the data provided in the ‘Molecular Information’ module, we develop the ‘Evolution & Comparative Genomics’ module. This module offers a suite of interactive tools for analyzing sequence conservation and evolutionary relationships, enabling researchers to interpret the functional diversification of IFN-I proteins and guide downstream experiments.

**Figure 6:**
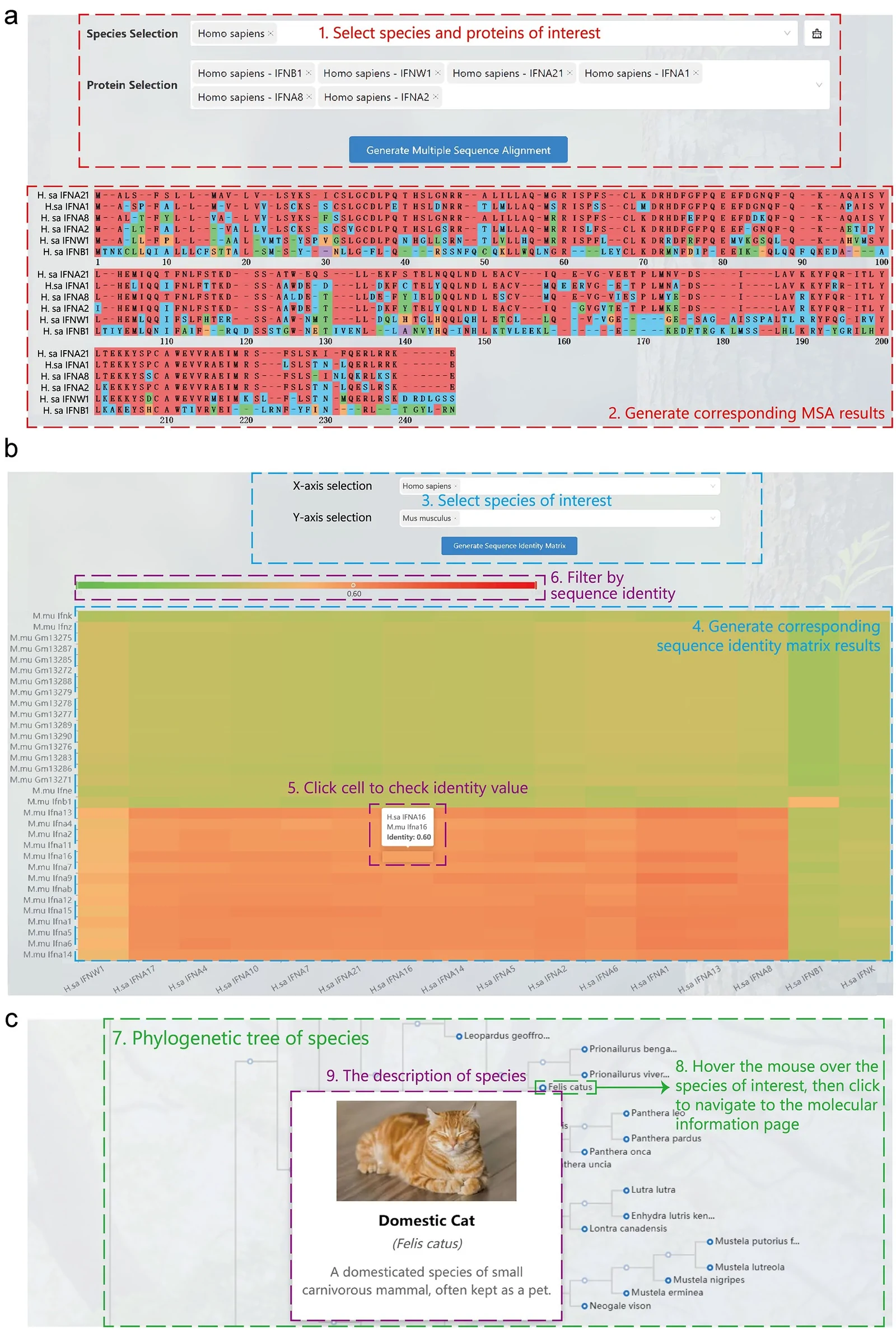
‘Evolution & Comparative genomic’ module. (a) The MSA tool. (b) The sequence identity analysis tool. (c) The phylogenetic tree of species involved in the study.

First, the ‘Multi-Sequence Alignment’ submodule supports the MSA analysis of IFN-I proteins within the database. Users can select and input multiple species of interest (e.g. *Homo sapiens*) and their corresponding IFN-I proteins (e.g. IFNB1) into the tool (Box 1 of Fig. 6a). Subsequently, based on the alignment results, amino acids and inserted gaps are rendered in various colors such as red, blue, and green (Box 2 of Fig. 6a). By revealing conserved domains and adaptively evolved regions within the IFN-I protein family, this submodule provides potential evolutionary evidence for the personalized design of IFN-I clinical therapies.

Additionally, we develop the ‘Sequence Identity Matrix’ submodule to provide cross-species sequence identity analysis. Similarly, users can select multiple species for both X and Y axis, and then obtain the sequence identity matrix of all IFN-I proteins between the selected axes (Boxes 3 and 4 of Fig. 6b). Users can also click on any individual cell within the matrix to retrieve the specific identity value between the two corresponding IFN-I proteins (Box 5 of Fig. 6b). We have incorporated a threshold bar feature into this component, which enables users to set an identity threshold and visualize the remaining cells that are greater than the threshold (Box 6 of Fig. 6b). The ‘Sequence Identity Matrix’ tool clearly demonstrates that the sequence identity among IFN-I orthologs is considerably greater than that among IFN-I paralogs, which is consistent with previous reports [58, 59], and helps users to understand the natural selection pressures driving the diversification of IFN-I subtypes.

Finally, within the ‘Phylogeny of Organisms’ submodule, we present a phylogenetic relationship diagram for the species involved in the study (Box 7 of Fig. 6c). By implementing a species introduction pop-up window, users can hover the mouse over a target species to obtain introductory information. This could assist users who are unfamiliar with taxonomy to gain a general overview of the species within this research (Boxes 8 and 9 of Fig. 6c). Furthermore, by clicking the Latin name of a specific species, the system redirects to the corresponding species detail page within the ‘Molecular Information’ module, which allows users to access their respective IFN-I gene and protein information.

## Case study: assessing the therapeutic potential of human IFN-α subtypes

**Figure 7:**
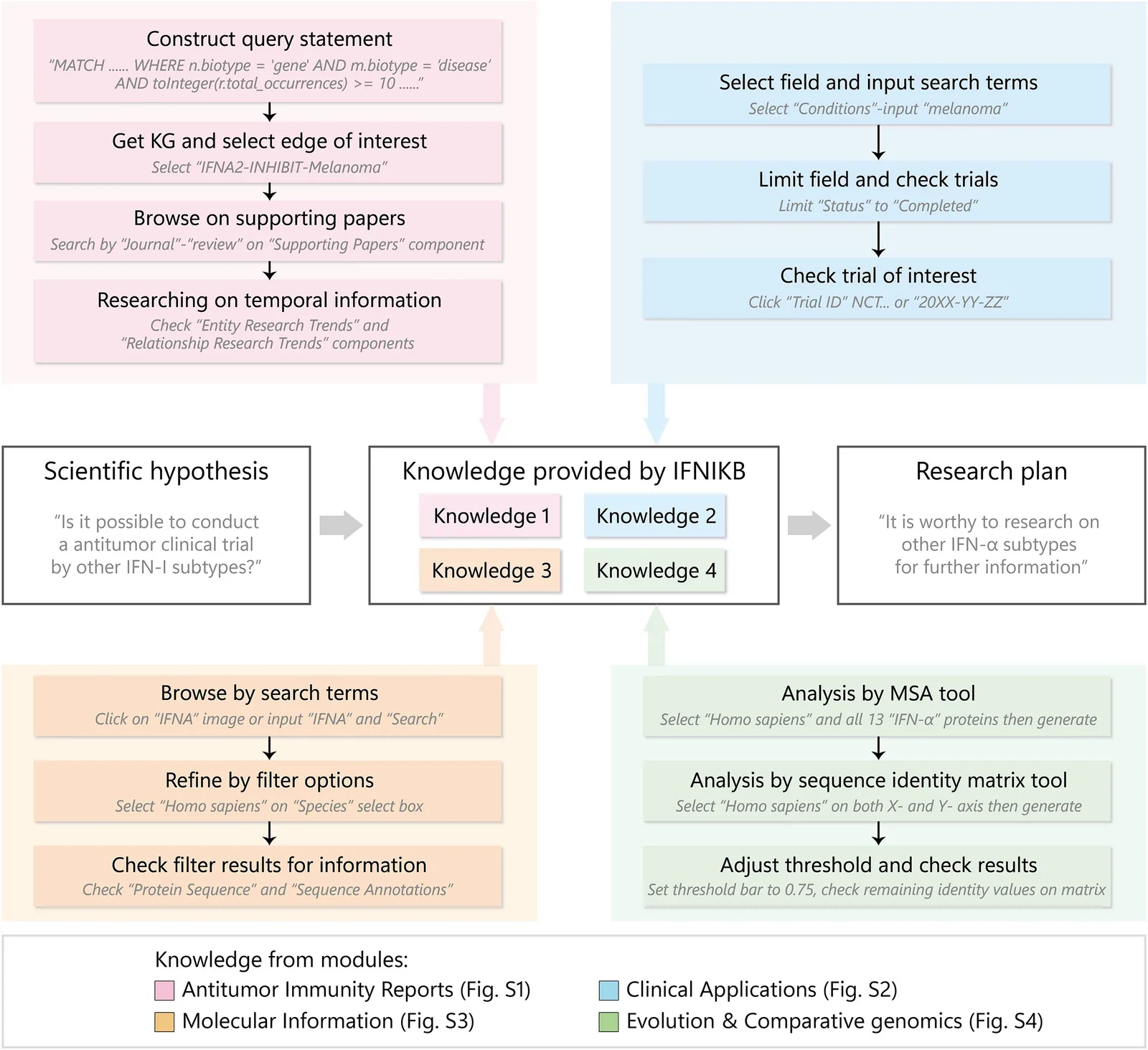
Knowledge derived from multiple IFNIKB modules facilitates IFN-I antitumour research.

We present a hypothetical case study to demonstrate the utility of IFNIKB’s four modules in generating a scientific hypothesis. The scenario involves a user investigating the antitumour potential of human IFN-I subtypes beyond the clinically approved IFN-α2 [2, 3, 16, 17]. The goal is to gather multi-dimensional evidence from literature, clinical trials, and genomics to rationalize further research (Fig. 7).

First, using the ‘Antitumour Immunity Reports’ module, the user confirms that the inhibitory effect of IFN-α2 on cancers like melanoma is extensively documented, supported by 140 publications and a long research history peaking in the early 2000s. However, reports on other IFN-I subtypes are notably scarce (Fig. S1). This literature-based finding is corroborated by the ‘Clinical Applications’ module, which identifies 63 completed trials involving IFN-α2 for melanoma, yet reveals a significant lack of clinical studies for other subtypes (Fig. S2).

To explore the molecular basis for investigating other subtypes, the user turns to the ‘Molecular Information’ module. This reveals that the human genome contains 13 functional IFNA genes, whose protein products (IFN-α subtypes) share highly conserved sequences, structures, and functional domains (Fig. S3). This observation is quantitatively validated in the ‘Evolution & Comparative Genomics’ module. Both multiple sequence alignment and a sequence identity matrix demonstrate that the amino acid identity among all human IFN-α subtypes exceeds 75% (Fig. S4).

By integrating these findings, the user concludes that while IFN-α2’s efficacy is well-established, numerous other human IFN-α subtypes with high sequence similarity remain largely unexplored both pre-clinically and clinically. IFNIKB efficiently provides this comprehensive evidence, establishing a strong scientific rationale to investigate the antitumour activity of these other IFN-α subtypes (e.g. receptor affinity, binding epitopes), and can be used to plan in vitro and animal experiments to validate IFN-I antitumour activity.

## Discussion

To date, recombinant protein IFN-α has been applied in the treatment of melanoma and renal cell carcinoma, while strategies for inducing endogenous IFN-α/β (such as ICD inducers, TLR agonists) and gene therapies are under continuous development [60]. However, despite nearly 70 years of IFN-I research history, no database that integrates the relevant knowledge of IFN-I is currently available.

For this reason, this study, on one hand, develops the first IFN-I-specific database, IFNIKB, which integrates literature reports, clinical trial records, as well as cross-species gene records and protein sequences. All data are sourced from authoritative public databases within the field, which guarantees that the data are up to date and originate from reliable sources (Fig. 1). On the other hand, we construct an automated workflow on knowledge query (Fig. 2) and knowledge reasoning (Fig. 3) for IFN-I antitumour immunity literature. As demonstrated in our case study (Fig. 7), users can employ the knowledge graph to map abstract biological or clinical terms related to IFN-I onto specific nodes and relationships, and explore the research history or related topics of the scientific questions through analysis components. In addition, we develop independent work modules to assist users in retrieving and filtering IFN-I clinical trial records (Fig. 4) and molecular information (Fig. 5) of interest, and carry out downstream analyses by various computational tools integrated within IFNIKB (Fig. 6). These features can assist researchers in acquiring and processing IFN-I knowledge, and facilitate the translation of IFN-I antitumour immunity research from basic discovery to clinical application.

In recent years, breakthrough biotechnologies have been increasingly applied to IFN-I-related research, and then they generate diverse IFN-I data. For example, during the COVID-19 pandemic, single-cell sequencing evidence revealed that the activation of IFN-I-related pathways, the suppression of ISG levels, and the production of anti-IFN-I autoantibodies are associated with the COVID-19 immune response and critical disease severity [61, 62]. Furthermore, protein structure predictive tools, exemplified by AlphaFold [63–65], have creatively transformed the physical and biological problem of crystal structure determination into a computer science problem, generating the high-throughput structural data according to the protein sequences. Specifically, the ‘AlphaFold Protein Structure Database’ has already indexed thousands of entries for the three-dimensional structures of IFN-I proteins [66]. Therefore, we will pay constant attention to new advancements and technologies in these fields, and incorporate diverse data into IFNIKB to attract more scientists to use the database in the distant future.

## Supplementary Material

baag053_Supplemental_File

## Data Availability

IFNIKB is publicly available at http://www.combio-lezhang.online/IFNIKB/home for interactive web-based access (current release: version v1.01). The resource is hosted on servers maintained by Prof. Le Zhang’s group at Sichuan University and will remain accessible for at least five years after publication. Updates are performed manually after upstream data releases, with a brief description of changes posted on the homepage. Server-side backups of each release are retained for recovery after system failure.
