# Correction: IFNIKB: a type I interferon database for antitumour immunity studies

**DOI:** 10.1093/database/baag063

**Published:** 2026-10-05

This is a correction to: Fubo Ma, Kang Li, Yangchao Yu, Lei Zhang, Liguo Zhang, Bing Li, Le Zhang, IFNIKB: a type I interferon database for antitumour immunity studies, *Database*, Volume 2026, 2026, baag053, https://doi.org/10.1093/database/baag053

Text in the title is emended to read “antitumour” and the same repeated error - “tumuor” - corrected to read “tumour” throughout the paper. The publisher apologises for this error introduced during production.

