# Supplementary material for: IFNIKB: a type I interferon database for antitumour immunity studies
Source: Database (Oxford). 2026 Sep 7;2026:baag053. doi: 10.1093/database/baag053 (PMC13548876; doi:10.1093/database/baag053)
Supplement: Supplementary file 1 [file baag053_supplemental_file.docx]

# Supplementary Information

**Supplementary Methods**

### Method S1. Detailed data collection and preprocessing protocols.

### Method S2. Detailed computational and visualization methods.

**Supplementary Tables**

**Table S1.** The terms and regular expressions used for data collection and preprocessing in the Methods section.

**Table S2.** Quality control of entities and relationships in the IFN-I antitumor immunity knowledge graph.

**Supplementary Figures**

**Figure S1.** Retrieving the current status of research reports on IFN-α2 treatment for melanoma via the “Antitumor Immunity Reports” module.

**Figure S2.** Retrieving records of completed clinical trials on IFN-I treatment for melanoma from the “Clinical Application” module.

**Figure S3.** Data from the “Molecular Information” module indicate the presence of up to 13 IFNA genes with similar sequences in humans.

**Figure S4.** Analysis results from the “Evolution & Comparative genomic” module indicate high sequence conservation among multiple IFN-α copies.

# Supplementary Methods

### Method S1. Detailed data collection and preprocessing protocols

#### S1.1 IFN-I antitumor immunity literatures

we collect 62,147 literature articles related to IFN-I antitumor immunity using query terms **(Table S1)** via the API from NCBI Entrez system. Subsequently, we employ filter terms and check terms **(Table S1)** to exclude literature unrelated to IFN-I topic.

#### S1.2 IFN-I clinical trials data

IFN-I clinical trials data are sourced from ClinicalTrials (CT) and EU Clinical Trials Register (EUCTR). For CT, data are obtained via query terms **(Table S1)** from the clinicaltrials.gov API. For EUCTR, as an available API is not provided, data are acquired using an automated web crawling framework (selenium (v4.23.1) and BeautifulSoup (v4.12.3)). All collected data are subsequently screened using filter terms and check terms **(Table S1)** to guarantee the relevance to IFN-I topic, and are followed by manual curation to standardize fields among different databases.

#### S1.3 IFN-I gene and protein data

We firstly search NCBI Gene by the term "interferon". Subsequently, entries corresponding to non-IFN-I genes are excluded by filter and check terms **(Table S1)**. Next, the API provided by the NCBI Entrez system is used to retrieve information for all identified IFN-I genes, such as species taxonomy, chromosomal location, gene location, corresponding protein sequences, and annotations, etc. This information is then converted to JSON format and stored by the species name as the filename.

### Method S2. Detailed computational and visualization methods

#### S2.1 Data computing

The IFN-I knowledge graph is constructed based on the 26,273 curated articles. We employ the API of PubTator3 to extract all biomedical entities (e.g., Gene, Disease, and Chemical) and semantic relationships (triplets) from the titles and abstracts. The graph is finalized through entity disambiguation and node merging steps to consolidate redundant information. The final graph is stored in a Neo4j (v4.3.1) database, enabling complex topological queries via the Cypher language.

For downstream analysis, time-series trends for both entities and relationships are generated by mapping paper IDs onto their publication dates (obtained via NCBI Entrez API) and computing their annual frequency distributions. Network topology analysis involves two key methods: “Node Centrality Analysis” is implemented using the degree centrality algorithm to identify highly connected nodes. “Community Detection Analysis” is performed using the Louvain algorithm, which consists of modularity optimization and community merging steps to identify densely connected subgraphs.

The “Evolution & Comparative genomics” module integrates several bioinformatics tools. The multiple sequence alignment (MSA) tool performs optimal alignment using the Needleman-Wunsch algorithm. The sequence identity matrix for selected IFN-I protein sequences is pre-computed by MEGA (v11.0). The phylogenetic relationships among all 654 species are based on a tree computed by TimeTree (v5.0).

#### S2.2 Data visualization

In this study, a responsive design for the website is implemented by HTML, CSS, and JavaScript. User interaction components are primarily based on the Ant Design (v5.0.3) component library, which provides standardized UI elements such as menus, tables, and buttons. The rendering of computational results, including time-series analysis, network topology analysis, sequence annotation, multiple sequence alignment (MSA), sequence identity matrix, and phylogenetic trees, are primarily implemented by Apache ECharts (v5.2.2).

# Supplementary Tables

**Table S1.** The terms and regular expressions used for data collection and preprocessing in the Methods section.

| **Section** | **Purpose** | **Terms and regular expressions** |
| --- | --- | --- |
| Methods 2.1.1 | Query for reports related to IFN-I in antitumor immunity | ((IFN-I OR type I interferon OR interferon alpha OR interferon-alpha OR IFN-alpha OR IFN-α OR IFN-alfa OR interferon alfa OR interferon-alfa OR interferon beta OR interferon-beta OR IFN-beta OR IFN-β OR interferon omega OR interferon-omega OR IFN-omega OR IFN-ω OR interferon kappa OR interferon-kappa OR IFN-kappa OR IFN-κ OR IFNAR1 OR IFNAR2 OR ISG) AND (antitumor immunity OR antitumor OR tumor OR cancer OR oncology)) |
| Methods 2.1.1 | Filter out irrelevant reports (only matching expressions in 2 but not in 1) | 1)  2)   |
| Methods 2.1.1 | Check the relevance of reports to IFN-I |  |
| Methods 2.1.2 | Query for clinical trials related to IFN-I | type I interferon OR IFN-I OR interferon alpha OR interferon-alpha OR IFN-alpha OR IFN-α OR IFN-alfa OR interferon alfa OR interferon-alfa OR interferon beta OR interferon-beta OR IFN-beta OR IFN-β OR interferon omega OR interferon-omega OR IFN-omega OR IFN-ω OR interferon kappa OR interferon-kappa OR IFN-kappa OR IFN-κ |
| Methods 2.1.2, 2.1.3 | Filter out irrelevant trials or genes | regulat, induc, stimulat, recept, type iii, respons, gamma, activ, double-strand, inhibit, relat, factor, type ii, lambda, lamda, bind, quality, ifnar, ifng, il, interleukin |
| Methods 2.1.2, 2.1.3 | Check the relevance of trials or genes to IFN-I | type I interferon, type I IFN, IFN-I, alpha, α, alfa, beta, β, omega, ω, kappa, κ |

**Table S2.** Quality control of entities and relationships in the IFN-I antitumor immunity knowledge graph.

| **Data Types** | **Biotypes** | **QC Metrics** | **Before QC*** | **After QC*** | **Ratio** |
| --- | --- | --- | --- | --- | --- |
| Entities | Chemical | MeSH | 3,235 | 3,222 | 99.6% |
|  | Disease | MeSH/OMIM | 3,062 | 3,058 | 99.6% |
|  | Gene | NCBI Gene | 4,391 | 4,360 | 99.3% |
|  | Species | NCBI Taxonomy | 1,114 | 1,097 | 98.5% |
|  | Cell line | Cellosaurus | 2,025 | 2,025 | 100% |
|  | Variant | LitVar | 704 | 704 | 100% |
|  | (Total) | - | 14,531 | 14,466 | 99.6% |
| Relationships | (Total) | - | 60,150 | 59,222 | 98.5% |

*Before QC (quality control) is the count in the knowledge-graph export prior to QC. After QC is the count retained after structural checks, vocabulary-based validation, and removal of invalid relationships.

# Supplementary Figures

**Figure S1. Retrieving the current status of research reports on IFN-α2 treatment for melanoma via the “Antitumor Immunity Reports” module.
(A)** Constructing the query statement by editing options within the "Visual Query Builder". **(B)** Obtaining information on the "IFN-α2-INHIBIT-Melanoma" triplet from the knowledge graph results and the pop-up window. **(C)** Retrieving review literature that supports the triplet. **(D)** Checking the publication years and trends of research related to the triplet via time-series analysis components.

**Figure S2. Retrieving records of completed clinical trials on IFN-I treatment for melanoma from the “Clinical Application” module.**

**Figure S3. Data from the “Molecular Information” module indicate the presence of up to 13 IFNA genes with similar sequences in humans.**

**(A)** Searching for the IFNA gene using the search bar or by clicking the corresponding image. **(B)** Restricting the results to “*Homo sapiens*” within the “Filter Options”. **(C)** Examining the detailed information on the gene card.

**Figure S4. Analysis results from the “Evolution & Comparative genomic” module indicate high sequence conservation among multiple IFN-α copies.**

**(A)** The MSA tool indicates that IFN-α subtypes are conserved across most functional regions. **(B)** The sequence identity matrix tool indicates that the sequence identity among IFN-α subtypes is greater than 0.75.
